# Intraoperative Ultrasonographic Imaging in Liver Surgery: A Review

**DOI:** 10.1155/1990/80639

**Published:** 1990

**Authors:** T. Ezaki, G. P. Stansby, K. E.F. Hobbs

**Affiliations:** Academic Department of Surgery Royal Free Hospital School of Medicine Pond Street London NW3 2QG UK


*HPB Surgery*, 1990, Vol. 3, pp. 1–4
Reprints available directly from the publisher
Photocopying permitted by license only

© 1990 Harwood Academic Publishers GmbH
Printed in the United Kingdom

### REVIEW ARTICLE

### INTRAOPERATIVE ULTRASONOGRAPHIC IMAGING IN LIVER SURGERY: A REVIEW

T. EZAKI, G.P. STANSBY and K.E.F. HOBBS
*Academic Department of Surgery, Royal Free Hospital School of Medicine, Pond Street, London. NW3 2QG, UK.*

KEY WORDS: Intraoperative ultrasonography, hepatic surgery, liver neoplasm

During the last decade liver surgery has become both safer and more widely practised[1–4]. Factors responsible include a greater appreciation of liver anatomy, improved anaesthesiology and perioperative care and technological advances such as the development of intraoperative ultrasonographic imaging, improved retractors, ultrasonic surgical aspirators, lasers and infrared and microwave coagulators. Hepatectomy can be performed without these facilities but increasingly surgeons are using them in order to perform hepatectomy more safely and precisely. Before the development of intraoperative ultrasound, inspection and palpation were the only way of assessing a hepatic lesion at laparotomy[5,6]. Now intraoperative ultrasound (US) is complementing this as more centres obtain both the necessary equipment and expertise.

The use of intraoperative US was first reported in the 1960's but the technique was not widely adopted because equipment was difficult to use and the images difficult to interpret. In the early 1980's, with refinements in equipment, intraoperative US finally became a useful diagnostic technique[7–9]. Using real time B-mode scanners it is possible to recognize the 3-dimensional nature of lesions and their relationship to the blood vessels of the liver as well as to identify small and previously undetectable lesions.

The method is essentially simple and involves placing a sterile probe directly on to the liver surface. Sterile jelly or normal saline solution can be applied to enhance sound transmission but this is usually unnecessary unless the liver surface is irregular as it is in macronodular cirrhosis. There exists a blind zone of 0.5 to 1 cm depth below the liver surface which can best be visualized by placing a water cushion approximately 2 cm in height between the liver and the probe[10]. Placement of a bulky probe in the uppermost portion of the liver may be handicapped by limited space. To overcome this, small probes can be used or the incision may need to be extended and rarely even a thoracotomy may be required[11]. However, retractors which clamp to the table and allow the costal margin to be forcibly elevated will allow adequate exposure in most cases.

Correspondence to: Dr T. Ezaki

Although a T-shaped probe is most commonly used, many other designs and types of probe are commercially available and it is advantageous to have a range of these available. A 5MHz transducer will usually give good image quality but if more penetration is required a 3MHz transducer can be used and occasionally, when questionable areas are found on an initial screening examination, near-field viewing with a 7.5MHz transducer is helpful[5].

Initial scanning involves identifying the hepatic and portal veins, gallbladder and hilus. A systematic examination is then performed in both sagittal and transverse planes[5,8,10,12–15]. The liver is delineated into posterior, anterior, medial and left lateral parts followed by subdivision into segments as described by Couinaud[10,15,16] The key to these segments is the arrangement of hepatic veins which in the normal liver are readily distinguishable from portal vein branches by visible transmitted pulsations. It is relatively easy to differentiate the anterior part of the right lobe, segments V and VIII, from the posterior part, segments VI and VII, by noting the position of the right hepatic vein. However, there is no clear division between segments V and VIII or VI and VII and each individual segment of the pair is identified separately by following the portal vein branch which runs into it. In the left lobe it may not be relevant to differentiate between segments II and III, unless the liver is cirrhotic with left lobe hypertrophy. In the caudate lobe (segment I) hepatic malignancy is rarely seen except in the case of cholangiocarcinoma extending into it[17].

Following identification of the vascular anatomy the hepatic lesion should be identified and there should then follow a careful search of the liver for additional lesions. Intraoperative US undoubtably provides the best assessment of the total number of lesions present in the liver and has a reported sensitivity of 78.5–100%[5,7,8,11,14,18–21]. This is better than any preoperatively performed imaging modality including preoperative US, angiography, computerised tomography (CT)[5,7,8,11,14,18–20] or lipiodol enhanced CT[21]. It is clearly the most useful modality for assessing lesions under 5cms diameter[11,18–20] since most lesions greater than 1cm diameter will be detected unless their echogenicity is similar to that of the surrounding liver[7,11,12,14,18–20]. As further improvements in equipment occur resolution can be expected to improve. Enhancement with carbon dioxide microbubbles injected into the hepatic artery has been described for preoperative US but has not been evaluated intraoperatively[22].

When lesions are discovered it is necessary to decide whether they are benign or malignant. Benign disease, especially focal steatosis and regenerative hyperplasia in a cirrhotic liver, is increasingly being detected by the widespread use of screening techniques[19,23]. However, whilst intraoperative US is highly sensitive in detecting such lesions it is less help in differentiating benign from malignant ones. Accuracy depends to a large extent on the texture of the surrounding liver and is usually highest in the normal liver. For metastatic liver lesions from colorectal carcinoma, the specificity of intraoperative US has been reported as being 94%, compared with 95.3% for preoperative US and 92.5% for preoperative CT[14], but when used in the detection of intrahepatic metastases of primary liver cancer, its specificity is significantly lower than that of the preoperative imaging modalities[7,21]. It may often only be possible to differentiate benign lesions from malignant ones by histologic examination of a sonographically guided intraoperative needle biopsy.

Once the whole liver has been scanned to identify secondary lesions the principal lesion can be more fully assessed. Intraoperative US can provide a demonstration of hepatic vascular anatomy in such a way as to be a helpful adjunct in determining both resectability and the extent of resection required[5,7,11,12–15,18,19,24]. Tumour thrombi in the portal vein can often be identified and their presence may have prognostic significance or contraindicate against resection. It has also helped in what was previously a major problem: the resection of, or the injection of ethanol or sclerosant into, small lesions which are both invisible and impalpable[5,11,21]. Approximately half of hepatocellular carcinomas less than 5cm will be impalpable and invisible at the time of surgery[20] and intraoperative US allows a limited resection to be performed in order to preserve residual liver function in a patient with chronic liver disease[25].

Since the introduction of screening techniques, including alpha-fetoprotein estimations and CT scanning, the earlier detection of hepatocellular carcinomas in high risk patients is possible[7,11,12,14,18–20]. This has lead to an increasing number of nonsegmental or wedge resections being carried out. Intraoperative US allows repeated assessment of the surgical margin during the resection of these small liver lesions[3,26]. For example, when a small liver cancer in a cirrhotic liver is situated in the bifurcation between the anterior and posterior branches of the right portal vein, where it may be invisible and impalpable, it is attractive to perform a limited hepatic resection in order to preserve residual liver function. The use of intraoperative US allows this to be done rather than attempting a very high risk formal right hepatic lobectomy. After mobilization of the entire liver, the relationship between the tumour and vessels is initially checked with ultrasound and this assessment is repeated frequently in order not to lose orientation. If the plane of resection is not clear, the fingers of the hand not holding the probe can be inserted and moved between the divided planes to act as a guide for orientation[7]. Such resection may also be aided by using the ultrasonic surgical aspirator with intermittent Pringle vascular clamping[27,28]. For deeply seated tumours it may be helpful to insert needles under ultrasound guidance to indicate the resection margin; alternatively dye can be injected into the parenchyma[7,11,27]. It is also possible to cannulate branches of the portal vein in the liver which can then be selectively ligated or occluded with a balloon catheter during the resection[28,29].

We believe that the use of intraoperative US will become as indispensable for liver surgeons as a stethoscope is for physicians. It is especially useful when considering surgery on small lesions or on the cirrhotic liver which has always presented special problems. The technique can be learned quickly, although accurate interpretation of the images will only come with experience. The only way to become proficient is to use it on every case until the normal anatomical structures can be clearly and easily identified. Its' use should become an essential component of the training of all liver surgeons.

### *References*

1. The liver cancer study group of Japan. (1987) Primary liver cancer in Japan Sixth report *Cancer*, **60**, 1400–11
2. Tang, Z.Y., Yu, Y.Q., Zhou, X.D., Ma, Z.C., Yang, R., Lu, J.Z., Lin, Z.Y., Yang, B.H. (1989) Surgery of small hepatocellular carcinoma. Analysis of 144 cases. *Cancer*, **64**, 536–41
3. Registry of hepatic metastases. (1988) Resection of the liver for colorectal carcinoma metastases: A multi-institutional study of indications for resection. *Surgery*, **103**, 278–87
4. Ryan, J., Faulkner, J. (1989) Liver resection without blood transfusion. *Am. J. Surg.*, **157**, 472–75

5. Parker, G.A., Lawrence, W., Horsley, J.S., Neifeld, J.P., Cook, D., Walsh, J., Brewer, W., Koretz, M.J. (1989) Intraoperative ultrasound of the liver affects operative decision making. *Ann. Surg.*, **209**, 569–77
6. Foster, J.H., Berman, M.M. (1977) Resection of metastatic tumors. In: Solid liver tumors Vol. XXII in the series, pp. 211–12 Major problems in clinical surgery, W.B. Saunders
7. Makuuchi, M., Hasegawa, H., Yamazaki, S., Takayasu, K., Moriyama, N. (1987) The use of operative ultrasound as an aid to liver resection in patients with hepatocellular carcinoma. *World J. Surg.*, **11**, 615–21
8. Rifkin, M.D., Rosato, F.E., Branch, H.M., Oster, J., Yang, S.L., Barbot, D.J., Marks, G.J. (1987) Intraoperative ultrasound of the liver. An important adjunctive tool for decision making in the operating room. *Ann. Surg.*, **205**, 466–72
9. Plainfosse, M.C., Merran, S. (1983) Work in progress: Intraoperative abdominal ultrasound. *Radiology*, **147**, 829–32
10. Castaing, D., Kunstlinger, F., Habib, N., Bismuth, H. (1985) Intraoperative ultrasonographic study of the liver. Methods and anatomic results. *Am. J. Surg.*, **149**, 676–82
11. Sheu, J.C., Lee, C.S., Sung, J.L., Chen, D.S., Yang, P.M., Lin, T.Y. (1985) Intraoperative hepatic ultrasonography. An indispensable procedure in resection of small hepatocellular carcinomas. *Surgery*, **97**, 97–103
12. Igawa, S., Sakai, K., Kinoshita, H., Hirohashi, K. (1985) Intraoperative sonography: Clinical usefulness in liver surgery. *Radiology*, **156**, 473–8
13. Bismuth, H., Castaing, D., Garden, J. (1987) The use of operative ultrasound in surgery of primary liver tumors. *World. J. Surg.*, **11**, 610–4
14. Machi, J., Isomoto, H., Yamashita, Y., Kurohiji, T., Schirouzu, K., Kakegawa, T. (1987) Intraoperative ultrasonography in screening for liver metastases from colorectal cancer: Comparative accuracy with traditional procedures. *Surgery*, **101**, 678–84
15. Traynor, O., Castaing, D., Bismuth, H. (1988) Peroperative ultrasonography in the surgery of hepatic tymors. *Br. J. Surg.*, **75**, 197–202
16. Couinaud, C. (1957) Le Foie: Etudes Anatomiques et Chirurgicales. Paris: Masson Paris
17. Mizumoto, R., Kawarada, Y., Suzuki, H. (1986) Surgical treatment of hilar carcinoma of the bile duct. *Surg. Gynecol. Obstet.*, **162**, 153–8
18. Castaing, D., Emond, J., Bismuth, H., Kunstlinger, F. (1986) Utility of operative ultrasound in the surgical management of liver tumors. *Ann. Surg.*, **204**, 600–5
19. Gozzetti, G., Mazziotti, A., Bolondi, L., Cavallari, A., Grigioni, W., Casanova, P., Bellusci, R., Villanacci, V., Labo, G. (1986) Intraoperative ultrasonography in surgery for liver tumors. *Surgery*, **99**, 523–30
20. Nagasue, N., Kohno, H., Chang, Y.C., Galizia, G., Hayashi, T., Yukaya, H., Nakamura, T. (1989) Intraoperative ultrasonography in resection of small hepatocellular carcinoma associated with cirrhosis. *Am. J. Surg.*, **158**, 40–2
21. Hayashi, N., Yamamoto, K., Tamaki, N., Shibata, T., Itoh, K., Fujisawa, I., Nakano, Y., Yamaoka, Y., Kobayashi, N., Mori, K., Ozawa, K., Torizuka, K. (1987) Metastic nodules of hepatocellular carcinoma: Detection with angiography, CT, and US. *Radiology*, **165**, 61–3
22. Matsuda, Y., Yabuuchi, I. (1986) Hepatic tumors: US contrast enhancement with CO2 microbubbles. *Radiology*, **161**, 701–705
23. Nagasue, N., Akamizu, H., Yukaya, H., Yuuki, I. (1984) Hepatocellular pseudotumor in the cirrhotic liver. Report of three cases. *Cancer*, **54**, 2487–94
24. Clarke, M.P., Kane, R.A., Steele, G. Jr., Hamilton, E.S., Ravikumar, T.S., Onik, G., Clouse, M.E. (1989) Prospective comparison of preoperative imaging and intraoperative ultrasonography in the detection of liver tumors. *Surgery*, **106**, 849–55
25. Kanematsu, T., Takenaka, K., Matsumata, T., Furuta, T., Sugimachi, K., Inokuchi, K. (1984) Limited hepatic resection effective for selected cirrhotic patients with primary liver cancer. *Ann. Surg.*, **199**, 51–66
26. Yoshida, Y., Kanematsu, T., Matsumata, T., Takenaka, K., Sugimachi, K. (1989) Surgical margin and recurrence after resection of hepatocellular carcinoma in patients with cirrhosis. Further evaluation of limited hepatic resection. *Ann. Surg.*, **209**, 297–301
27. Makuuchi, M., Hasegawa, H., Yamazaki, S. (1985) Ultrasonically guided subsegmentectomy. *Surg. Gynecol. Obstet.*, **161**, 346–50
28. Shimamura, Y., Gunven, P., Takenaka, Y., Shimizu, H., Akimoto, H., Shima, Y., Arima, K., Takahashi, A., Kitaya, T., Matsuyama, T., Hasegawa, H. (1986) Selective portal branch occlusion by balloon catheter during liver resection. *Surgery*, **100**, 938–941
29. Castaing, D., Garden, J., Bismuth, H. (1989) Segmental liver resection using ultrasound-guided selective portal venous occlusion. *Ann. Surg.*, **210**, 20–3

